# Emerging Role of Caveolin-1 in GLP-1 Action

**DOI:** 10.3389/fendo.2021.668012

**Published:** 2021-04-14

**Authors:** Alessandra Puddu, Davide Maggi

**Affiliations:** Department of Internal Medicine and Medical Specialties, University of Genoa, Genoa, Italy

**Keywords:** glucagon-like peptide-1, caveolin-1, GLP-1 receptor, G proteins, β-arrestin-1

## Abstract

Glucagon-like peptide-1 (GLP-1) is a gut hormone mainly produced in the intestinal epithelial endocrine L cells, involved in maintaining glucose homeostasis. The use of GLP-1 analogous and dipeptidyl peptidase-IV (DPP-IV) inhibitors is well-established in Type 2 Diabetes. The efficacy of these therapies is related to the activation of GLP-1 receptor (GLP-1R), which is widely expressed in several tissues. Therefore, GLP-1 is of great clinical interest not only for its actions at the level of the beta cells, but also for the extra-pancreatic effects. Activation of GLP-1R results in intracellular signaling that is regulated by availability of downstream molecules and receptor internalization. It has been shown that GLP-1R co-localizes with caveolin-1, the main component of caveolae, small invagination of the plasma membrane, which are involved in controlling receptor activity by assembling signaling complexes and regulating receptor trafficking. The aim of this review is to outline the important role of caveolin-1 in mediating biological effects of GLP-1 and its analogous.

## Introduction

The biological effects of the Glucagon-like peptide-1 (GLP-1) are mediated by binding to its receptor, GLP-1R, a specific seven-transmembrane receptor guanine nucleotide-binding protein (G-protein) coupled receptor (GPCR) (1). GLP1 binding to GLP-1R leads to activation of intracellular signaling pathways that take part to the regulation of glucose homeostasis. The GLP-1R localizes in caveolae (2), a subset of lipid rafts on the plasma membrane structured as flask-shaped invagination of about 50 to 100 nm, composed of Caveolins and Cavins (3, 4). Caveolae regulate several cellular processes, including protein endocytosis, intracellular trafficking, cholesterol homeostasis, and signal transduction (5–7). In particular, Caveolin 1 (Cav-1) binds several signaling and structural proteins through the caveolin-binding motifs, a conserved sequence enriched with aromatic residues [ΦXΦXXXXΦ, ΦXXXXΦXXΦ, and ΦXΦXXXXΦXXΦ (Φ = aromatic residue, X = any amino acid)], found in a lot of proteins, including the GLP-1 receptor.

The direct interaction between GLP-1R and Cav-1 regulates the proper targeting of the GLP-1R to the plasma membrane, the receptor trafficking, and the activation of the intracellular signaling pathway. However, Cav-1 is also a multifunctional platform able to recruit several signaling molecules.

In this review we highlight the importance of Cav-1 in mediating GLP-1 action. Moreover, we speculated about the involvement of Cav-1 in regulating GLP-1 signaling at the level of the G proteins, showing that Cav-1 might modulate signal transduction by influencing not only the trafficking of GLP-1R, but also that of the signaling proteins. Finally, we hypothesize that Cav-1 may regulate GLP-1 action binding both GLP-1R and β-arrestin-1, and modulating the activity of Dipeptidyl Peptidase (DPP)-IV.

## Caveolin-1

The principal protein of caveolae is Cav-1, a 22- to 24-kDa integral membrane protein with a hairpin-like conformation ubiquitously expressed in many different tissues, except striated muscle in which Caveolin 3 is highly expressed (6, 8). The hairpin loop is transmembrane, whereas the amino- and carboxy-terminal domains are oriented towards the cytoplasm. In particular, the juxtamembrane domain in the N-terminal region of the protein acts as a scaffolding protein and, besides driving caveolae formation through heterooligomeric complex with Caveolin-2 and PTRF-cavin (9), interacts with a variety of signaling molecules such as G-proteins, H-Ras, Fyn, Erk-2, Src family tyrosine kinases, and so on (10, 11). Moreover, Cav-1 directly interact with insulin and IGF1 receptor and their principal substrate IRS-1 supporting a role in metabolic regulation (12, 13).

## GLP-1 and its Receptor

GLP-1 is an incretin hormone derived from the proglucagon gene and secreted by the intestinal L cell in response to food ingestion to maintain glucose homeostasis (14). The secretion of GLP-1 is reduced in Type 2 Diabetes Patients (15–17). Therefore, therapies with GLP-1 receptor agonists and DPP-4 inhibitors are largely employed to restore incretin action in T2D. The improvement of pancreatic beta cell dysfunction and the protective role of GLP-1 against oxidative stress has been described both *in vitro* and *in vivo* (17–21). These biological effects of GLP-1 are selectively mediated by activation of GLP-1R, that leads to various intracellular signaling pathways mainly described in pancreatic beta-cells (22). Briefly, GLP-1 binding to its receptor triggers G-protein activation which leads to cAMP production, calcium mobilization and phosphorylation of extracellular signal-regulated kinases (ERK). In addition, GLP-1R is expressed also in peripheral tissues, including the central and peripheral nervous systems, heart, kidney, lung, gastrointestinal tract and retinal pigment epithelium (23, 24). Therefore, the great clinical interest on GLP-1 for the management of type 2 diabetes is due not only to its actions at the level of the beta cells, as well in the peripheral tissues.

## Interaction Between GLP-1R and Caveolin-1

The GLP-1R sequence contains a classical caveolin-1 binding motif within the second intracellular loop (247-EGV**Y**L**Y**TLLA**F**SV**F**-260) (Uniprot) (1, 25). The first evidence that GLP-1R directly interacts with Cav-1 has been reported by Syme et al. in 2006 (2). They showed that Cav-1 immunoprecipitated with GLP-1R, and that, on the contrary, mutation of the two tyrosine residues Y250 and Y252 to alanine in the GLP-1R amino acidic sequence abrogated the interaction of GLP-1R with Cav-1.

Direct interaction with Cav-1 is required for internalization of receptors in caveolae and also for trafficking of GLP-1R (26–28). For instance, GLP-1R containing Y250/252A mutations is trapped in intracellular compartments, and not localized on the cell surface (2). On the other contrary, GLP-1R is not internalized after agonist stimulation in cells expressing P132L-Cav-1, a mutated form of Cav-1 that results in misfolded oligomers which accumulate within the Golgi complex (29), and in cells treated with caveolae inhibitors (2).

## Caveolin-1 and GLP-1 Signaling

The subcellular localization of a receptor is an important mechanism that regulates signaling specificity. Consequently, in case of receptors containing GLP-1 Y250/252A mutations, which prevent the localization of GLP-1R on the plasmamembrane and the binding of GLP-1, the intracellular signaling is lost (2). On the contrary, defective internalization may lead to sustained activation of GLP-1R–mediated signaling (30). Therefore, the interaction between GLP-1R and Cav-1 is necessary not only for receptor trafficking to the cell membrane, but also for activation of the intracellular signaling pathway.

The fate of a receptor after activation is another important mechanism to control its signaling capacity: GLP-1R undergoes agonist-mediated endocytosis, which may lead either to recycle the receptor back to the plasma membrane or to degradative pathway (31–33). Considering that Cav-1 is required for internalization of GLP-1R after agonist stimulation (34), it is conceivable that Cav-1 may affect also the fate of GLP-1R determining its recycling or degradation.

GLP-1R internalization is also important for the spatiotemporal control of signaling. GLP-1R agonists exerted different effects on regulatory mechanisms that control the duration of receptor activation, such as desensitization and internalization (32). In particular, GLP-1 and exendin-4 are 10-fold more potent to cause GLP-1R internalization than liraglutide, but GLP-1 causes the receptor to recycle two to three times faster than when stimulated with exendin-4 or liraglutide (32). The rate at which GLP-1 and its analogs induce GLP-1R internalization may be affected by Cav-1. Indeed, Cav-1 selectively recruits and organizes proteins and lipids in membranes, therefore the different effects of GLP-1 agonists on GLP-1R activation may be due to the various compartmentalization of signaling molecules in caveolae. For instance, caveolae regulate many GPCR signaling pathways through a selective compartmentalization of G proteins, and their downstream targets in membrane microdomains (35, 36). In pancreatic beta cells GLP-1 can activate both Gαs and Gαq subunits. The Gαs pathway activates adenylyl cyclase leading to increase formation of cAMP; whereas the Gαq pathway leads to increase cytoplasmic concentration of Ca^2+^ (37). It is well documented that Gαs and adenylyl cyclase are localized in caveolae (38, 39), and that Gαs is quickly internalized after activation (40). Gαs internalization attenuates Gαs/adenylyl cyclase signaling and depends on Cav-1 (41, 42). Depletion of Cav-1 prevented agonist-induced internalization of Gαs in C6 glioma cells, with consequent increment of the Gαs/adenylyl cyclase signaling (42). On the other hand, depletion of Cav-1 inhibits Gαq-mediated signaling in C6 cells (43). It has been reported that Gαq is associated with Cav-1 at both plasma membrane and cytosolic level (44), and that Cav-1 binds preferentially to Gαq in its activated state, thus prolonging its activation (45). Interestingly, the activation of the Gαq pathway is required for agonist-induced GLP-1R internalization (34). Taken together these evidence suggest that Cav-1 may regulate GLP-1 action by controlling the duration of G-protein signals.

Activation of Gαs and Gαq pathways results in the translocation and exocytosis of insulin-containing secretory granules in pancreatic beta cells by increasing cytoplasmic concentration of Ca^2+^ through 2 mechanisms: closure of ATP-sensitive potassium channel (KATP) which leads to calcium influx *via* voltage-gated Ca^2+^ channels; and release of Ca^2+^ from intracellular Ca^2+^ stores (37). It has been reported that the KATP channel activity depends on the spatial organization of signaling pathways, requiring co-localization with adenylyl cyclase, and that the integrity of caveolae is important for adenylyl cyclase-mediated channel modulation (46). We previously demonstrated that the Kir6.2 subunit of the KATP channels is associated to Cav-1 in the pancreatic beta cell line βTC-6, and that depletion of Cav-1 reduced glucose induced insulin secretion (47). These evidence support the hypothesis that Cav-1 is also essential in GLP-1–induced insulin secretion by maintaining the correct regulation of KATP channels.

GLP-1 action is also mediated by β-arrestin-1 (48), a scaffolding protein that mediates receptor desensitization, receptor internalization, and links GPCRs to downstream pathways (49). Indeed, β-arrestin-1 knockdown attenuated GLP-1 signaling and impaired both glucose- and GLP-1–induced insulin secretion in INS-1 pancreatic beta cells (48). Interestingly, β-arrestin-1 directly interacts with both GLP-1R and Cav-1 (48, 50), therefore Cav-1, GLP-1R and β-arrestin-1 may form a microdomain implicated in regulating GLP-1 action. Depletion of β-arrestin-1 did not affect GLP-1R agonist-induced GLP-1 R internalization (48), suggesting that β-arrestin-1 acts downstream to Cav-1.

GLP-1 exerts also proliferative and antiapoptotic, effects activating ERK and AKT signaling pathways (17, 51). Interestingly, Cav-1 depletion protects pancreatic β cells against palmitate-induced dysfunction and apoptosis enhancing activities of Akt and ERK1/2. Considering that Cav-1 is required for internalization of GLP-1R, and that inhibition of GLP-1R internalization prolongs ERK activity (30), these findings suggest that Cav-1 depletion may affect ERK activity by regulating cellular trafficking of GLP-1R. On the other hand, β-arrestin-1 depletion decreased ERK phosphorylation (48), confirming that β-arrestin-1 acts downstream to Cav-1.

### CAVEOLIN--1 and DPP-IV

It is well known that GLP-1 has a short plasma half-life (1–7 min) due to quickly degradation by Dipeptidyl Peptidase (DPP)-IV/CD26, which is an integral membrane protein widely expressed on cell surfaces and, after cleavage, present in the circulation as “soluble” DPP-IV” (52). Interestingly, Cav-1 directly interacts with DPP-IV by binding to its serine catalytic site (53, 54). Furthermore, gene knockdown of Cav-1 suppressed the anti-inflammatory effects of the DPP-4 inhibitor teneligliptin in human monocyte/macrophage U937, showing that teneligliptin needs to bind Cav-1 to exert its effects (54). These evidence suggest that Cav-1 may affect lifespan of GLP-1 by regulating the activity of DPP-IV and of its inhibitors.

## Conclusions

**Figure 1:**
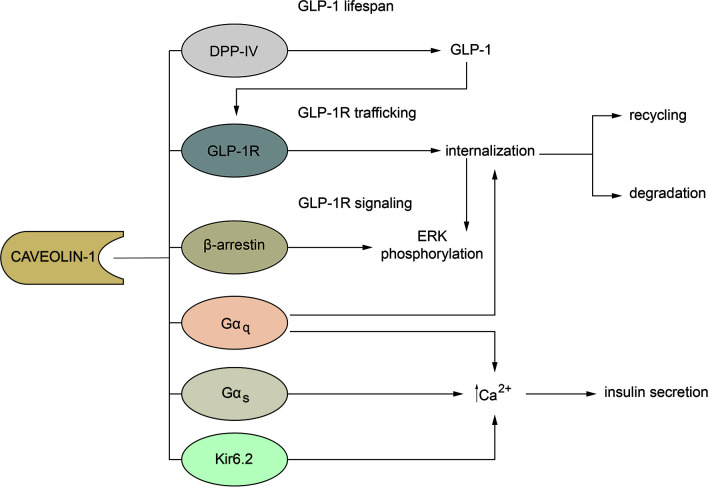
Main mechanisms through which Cav-1 regulates GLP-1 action.

Although it is well recognized that Cav-1 is involved in all the steps that regulate GLP-1 function, these aspects are not fully elucidated. Considering the knowledge in the literature, we can conclude that: 1) the interaction between GLP-1R and Cav-1 is necessary not only for receptor trafficking to the cell membrane, but also for activation of the intracellular signaling pathway; 2) Cav-1 may affect the fate of GLP-1R; 3) Cav-1 may regulate GLP-1 action by controlling the duration of G-proteins signals; 4) Cav-1 may be a physical link between GLP-1R and β-arrestin-1; 5) Cav-1 may affect lifespan of GLP-1 (**Figure 1**).

## Author Contributions

All authors listed have made a substantial, direct, and intellectual contribution to the work and approved it for publication.

## Conflict of Interest

The authors declare that the research was conducted in the absence of any commercial or financial relationships that could be construed as a potential conflict of interest.
